# ukbREST: efficient and streamlined data access for reproducible research in large biobanks

**DOI:** 10.1093/bioinformatics/bty925

**Published:** 2018-11-05

**Authors:** Milton Pividori, Hae Kyung Im

**Affiliations:** 1 Department of Medicine, Section of Genetic Medicine, The University of Chicago, Chicago, IL, USA; 2 Center for Translational Data Science, The University of Chicago, Chicago, IL, USA

## Abstract

**Summary:**

Large biobanks, such as UK Biobank with half a million participants, are changing the scale and availability of genotypic and phenotypic data for researchers to ask fundamental questions about the biology of health and disease. The breadth of the UK Biobank data is enabling discoveries at an unprecedented pace. However, this size and complexity pose new challenges to investigators who need to keep the accruing data up to date, comply with potential consent changes, and efficiently and reproducibly extract subsets of the data to answer specific scientific questions. Here we propose a tool called ukbREST designed for the UK Biobank study (easily extensible to other biobanks), which allows authorized users to efficiently retrieve phenotypic and genetic data. It exposes a REST API that makes data highly accessible inside a private and secure network, allowing the data specification in a human readable text format easily shareable with other researchers. These characteristics make ukbREST an important tool to make biobank’s valuable data more readily accessible to the research community and facilitate reproducibility of the analysis, a key aspect of science.

**Availability and implementation:**

It is implemented in Python using the Flask-RESTful framework for the API, and it is under the MIT license. It works with PostgreSQL and a Docker image is available for easy deployment. The source code and documentation is available in Github: https://github.com/hakyimlab/ukbrest.

## 1 Introduction

Large-scale biobanks provide invaluable resources to the scientific community to investigate the causes of disease (Gaziano *et al.*, 2016; Kvale *et al.*, 2015; Sudlow *et al.*, 2015). UK Biobank, the most mature of them, is a prospective study of the health of individuals based in the UK (Bycroft *et al.*, 2018). It has collected and continues to collect extensive genetic, environmental, lifestyle, medical history, physical and imaging measures and biological samples of ∼500 000 middle-aged individuals recruited between 2006 and 2010. All the data are openly shared with all *bona fide* investigators who want to conduct health-related research for the benefit of the public.

In the UK Biobank, a data-field is identified with an ID followed by two additional indices: *instance* and *array.* An instance indicates the time at which the data was collected (typically, instance 0 corresponds to the initial visit and larger numbers are used for subsequent visits). Array indicates one of the multiple values a categorical variable can take. For example, data-field 20002 indicates self-reported diseases, where each disease has a specific code assigned. An individual who reported suffering from asthma (coded as 1111) and hypertension (1065) during the initial visit, and diabetes (1220) in the second visit would have 20002_0_0 = 1111, 20002_0_1 = 1065 and 20002_1_0 = 1220. A data-field belongs to a particular category, can have different types (text, continuous, integer, date, categorical, among others), be single or multi-valued and in the case of disease data, it also has a hierarchical structure. For example, *deep venous thrombosis* and *pulmonary embolism* both belong to the parent group *venous thromboembolic disease* and cases of the latter can be reported with any of the terms.

Given this complexity, maintenance and reproducible phenotype and covariate extraction can be challenging. To address these problems we developed *ukbREST*, a user friendly tool that enables researchers to efficiently load the UK Biobank data into an SQL database, query any data-field and reproducibly document the phenotypes derived.

## 2 Deployment, usage and security

### 2.1 Setup

**Fig. 1.**
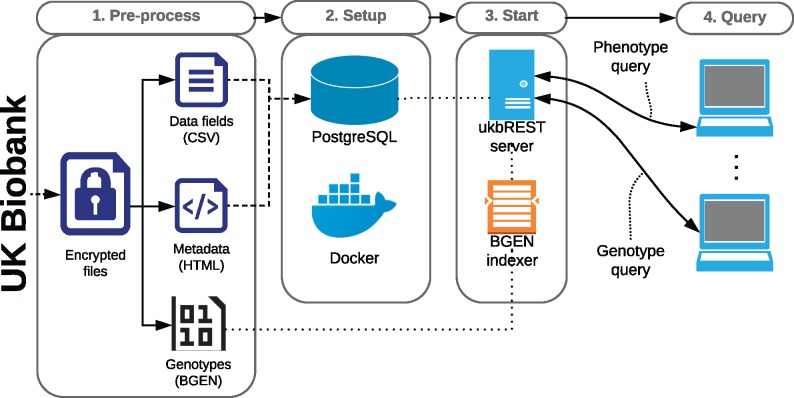
Workflow and components of ukbREST. After downloading the data the user will pre-process it following standard UK Biobank procedures. Next, he/she will use our Docker image which will automatically setup PostgreSQL, load the data and start the server. Finally, the server will be ready to accept queries

The steps needed to setup ukbREST as well as the workflow and components involved are shown in Figure 1. The first step needed to get ukbREST installed and running consists in *pre-processing* the encrypted data files obtained from UK Biobank following the standard procedures (using tools like ukbconv), producing files in CSV and HTML formats for phenotypes and BGEN for genotypes. In the second step, a PostgreSQL database is *setup* and phenotype data are loaded into it. This step is very easily performed by using containerization technology and scripts already provided by ukbREST. In the third step, the ukbREST server is *started* and now it is ready to receive *queries* by any authorized user using the REST API (with authentication and encryption capabilities).

### 2.2 Reproducible phenotype specification

The user has the option to request raw data-fields using simple standard tools like curl or more complex derived phenotypes by specifying a complete query file in a human readable YAML format. The YAML file requires two sections: (i) the samples filters, which specify a set of global conditions to select a subset of the individuals, and (ii) the data specification, which is used to get simple data (such as sex or height) or more complex phenotypes. An example of YAML file is shown below:

 1 **samples_filters**:

 2  - cin_white_british_ancestry_subset_0_0 = 1

 3  - eid not in (select eid from withdrawals)

 4

 5 **data**:

 6  sex: c31_0_0

 7  smoking_status: >

 8   coalesce (

 9    nullifneg(c20116_2_0), nullifneg(c20116_1_0),

 10    nullifneg(c20116_0_0)

 11   )

 12  asthma:

 13   case_control:

 14    20002:

 15     coding: 1111

 16    41202:

 17  coding: [J45, J450, J451, J458, J459]

Here the sample filters (line 1) include only white individuals of British descent and exclude those who have withdrawn their consent. The data section (line 5) specifies three columns: the first is sex, where it just selects data-field 31. The second column, named smoking_status, uses an SQL statement to merge all the information present in data-field 20116 from all instances, selecting the first one with non-missing data, prioritizing the latest information available (instance 2); a special function (nullifneg) is used to filter out missing data (coded with negative values). Finally, the asthma column uses a special feature that allows to easily specify cases and controls and merge data from different data-fields. In this case, cases are defined as either those who have self-reported asthma (which is coded with 1111 in data-field 20002) or have a hospital record with an asthma-related ICD10 code (data-field 41202); controls are all the rest that do not meet these criteria. The YAML file allows to specify more complex queries by, e.g. directly writing SQL code or defining aliases to avoid duplication. We also provide a special function that aggregates all children nodes of a disease code within a hierarchical structure.

We have posted sample YAML files (https://github.com/hakyimlab/ukbrest/wiki/YAML-examples-of-real-use-cases) that the users can use as a starting point and further edit for their needs. We also encourage users to share the YAML files for the benefit of the community.

### 2.3 Security

By default, ukbREST is only reachable from the computer/server where it was installed. No one from the network will be able to make any queries other than the user from the computer where ukbREST is running. In the online documentation we provide further instructions for more advanced network setups, including user authentication and encryption.
